# Childhood adversity, pubertal timing and self-harm: a longitudinal cohort study

**DOI:** 10.1017/S0033291721000611

**Published:** 2022-12

**Authors:** Abigail Emma Russell, Carol Joinson, Elystan Roberts, Jon Heron, Tamsin Ford, David Gunnell, Paul Moran, Caroline Relton, Matthew Suderman, Becky Mars

**Affiliations:** 1 Children and Young People's Mental Health Research Collaboration, University of Exeter College of Medicine and Health, Exeter, UK; 2 Centre for Academic Mental Health, Population Health Sciences, University of Bristol Medical School, Bristol, UK; 3 Department of Psychiatry, University of Cambridge, Cambridge, UK; 4 NIHR Biomedical Research Centre at the University Hospitals Bristol NHS Foundation Trust and the University of Bristol, Bristol, UK; 5 MRC Integrative Epidemiology Unit, Population Health Sciences, University of Bristol Medical School, Bristol, UK

**Keywords:** ACEs, adversity, ALSPAC, mediation, menarche, peak height velocity, puberty, self-harm, suicide attempt

## Abstract

**Background:**

The occurrence of early childhood adversity is strongly linked to later self-harm, but there is poor understanding of how this distal risk factor might influence later behaviours. One possible mechanism is through an earlier onset of puberty in children exposed to adversity, since early puberty is associated with an increased risk of adolescent self-harm. We investigated whether early pubertal timing mediates the association between childhood adversity and later self-harm.

**Methods:**

Participants were 6698 young people from a UK population-based birth cohort (ALSPAC). We measured exposure to nine types of adversity from 0 to 9 years old, and self-harm when participants were aged 16 and 21 years. Pubertal timing measures were age at peak height velocity (aPHV – males and females) and age at menarche (AAM). We used generalised structural equation modelling for analyses.

**Results:**

For every additional type of adversity; participants had an average 12–14% increased risk of self-harm by 16. Relative risk (RR) estimates were stronger for direct effects when outcomes were self-harm with suicidal intent. There was no evidence that earlier pubertal timing mediated the association between adversity and self-harm [indirect effect RR 1.00, 95% confidence interval (CI) 1.00–1.00 for aPHV and RR 1.00, 95% CI 1.00–1.01 for AAM].

**Conclusions:**

A cumulative measure of exposure to multiple types of adversity does not confer an increased risk of self-harm via early pubertal timing, however both childhood adversity and early puberty are risk factors for later self-harm. Research identifying mechanisms underlying the link between childhood adversity and later self-harm is needed to inform interventions.

## Introduction

Self-harm, defined in this study as intentionally hurting oneself in any way with or without suicidal intent, is a global public health concern. There is evidence that rates of self-harm are rising, and it is one of the strongest predictors of later suicide (Hawton et al., 2003; McManus et al., 2019). Understanding the causal antecedents of self-harm is important in reducing risk and developing effective treatments. One of the strongest risk factors for self-harm is experiencing adversity early in life (Björkenstam, Kosidou, & Björkenstam, 2016, 2017; Cha et al., 2018; Hughes et al., 2017). Adverse childhood experiences (ACEs) are commonly conceptualised as indicators of abuse, child maltreatment or household dysfunction (Björkenstam et al., 2016, 2017; Dube, Felitti, Dong, Giles, & Anda, 2003), including sexual, physical and emotional abuse as well as parental psychopathology and witnessing domestic violence. Studies exploring the role of ACEs in relation to health outcomes commonly consider the multiple types of adversity an individual has experienced, rather than focussing on individual adversities (Enns et al., 2006). Because adversities rarely occur in isolation and some, such as sexual abuse, are less common, it is difficult to disentangle the role of an individual adversity on child outcomes in population-based samples.

Understanding the pathways that link ACEs to an increased risk of later self-harm could improve the identification of those most at risk and may lead to novel intervention targets. However, the mechanisms linking ACEs to self-harm in adolescence and adulthood are unclear (Cha et al., 2018). One possible mechanism is through an earlier onset of puberty in children exposed to ACEs, since early puberty is associated with an increased risk of self-harm in adolescence and young adulthood in both sexes (Cha et al., 2018; Deng et al., 2011; Larsson & Sund, 2008; Michaud, Suris, & Deppen, 2006; Roberts, Fraser, Gunnell, Joinson, & Mars, 2020; Wichstrøm, 2000). There is some evidence that childhood adversity is associated with an earlier age at onset of puberty in both females and males (Henrichs et al., 2014; Lei, Beach, & Simons, 2018; Mendle, 2014) but empirical findings are mixed (Zhang, Zhang, & Sun, 2019). Adverse experiences that have been found to be associated with early pubertal timing in females include physical abuse, sexual abuse, parental mental illness, parent drinking, father absence, parental unemployment, frequent relocation and family violence (Arim, Tramonte, Shapka, Dahinten, & Willms, 2011; Barrios et al., 2015; Bogaert, 2005; Boynton-Jarrett et al., 2013; Boynton-Jarrett & Harville, 2012; Clutterbuck, Adams, & Nettle, 2015; Henrichs et al., 2014; Hulanicka, Gronkiewicz, & Koniarek, 2001; Kelly, Zilanawala, Sacker, Hiatt, & Viner, 2017; Li, Denholm, & Power, 2014; Magnus et al., 2018; Romans, Martin, Gendall, & Herbison, 2003). However, studies have also reported a later age at menarche (AAM) for females affected by abuse, domestic violence, family conflict and maternal alcohol abuse (Boynton-Jarrett et al., 2013; Boynton-Jarrett & Harville, 2012; Li et al., 2014). In males, there is evidence that low parental education and father absence (Arim et al., 2011; Bogaert, 2005) are associated with earlier age of puberty.

### Mechanisms and intervention potential

The developmental origins of health and disease theoretical framework proposes that environmental stress impacts the hypothalamic–pituitary–adrenal (HPA) axis, leading to elevated levels of stress hormones that are involved in the regulation of pubertal timing (Belsky & Shalev, 2016). HPA axis dysregulation and other stress-related physiological and epigenetic pathways have also been found to be associated with a greater risk of suicide and self-harm (Berardelli et al., 2020; Turecki & Brent, 2016).

Understanding if there are biological pathways that underpin the relationship between childhood adversity and self-harm will increase our understanding of factors that contribute to the aetiology of self-harm. Understanding this, and the relative contributions of biological and psychological mediators will highlight potential targets for psychosocial interventions. For example, early pubertal timing may lead to self-harm via adolescent social maladjustment, where those with early pubertal timing may associate with older peers, engaging in more risky behaviours such as alcohol use sooner than their same-age peers, increasing risk of self-harm (Patton et al., 2007). Interventions could promote adaptive coping skills, social negotiation skills, assertiveness and emotion regulation. The timing and quality of puberty education in schools (an ideal setting for such an intervention) could be improved to ensure those who have an earlier puberty are supported adequately; positive psychosocial adjustment at this point could reduce risk of later self-harm. Psychosocial interventions may also intervene on the pathways from ACEs to pubertal timing by using stress-tolerance techniques or by supporting families and children to maintain good mental health in the face of adversity and to reduce children's experiences of adversity.

Given these hypothesised common causal mechanisms from adversity to earlier onset of puberty and self-harm, we investigated whether pubertal timing lies on the causal pathway from ACEs to self-harm. Specifically, we aimed to assess whether pubertal timing mediated the association between the cumulative types of adversity a young person has experienced and risk of self-harm in adolescence and young adulthood, using data from a longitudinal birth cohort. We hypothesised that ACEs would be associated with earlier pubertal timing (measured as a continuous variable), and that pubertal timing would lie on the causal pathway between adversity and self-harm.

### The current study

To our knowledge, there is no published research investigating whether early pubertal timing is a mediator of the relationship between ACEs and risk of self-harm. We used data from a large UK birth cohort to investigate the relationship between ACEs (assessed from birth to 9 years), pubertal timing and self-harm, and we specifically examined whether early pubertal timing is a mediator of the relationship between ACEs and self-harm at 16 and 21 years.

## Methods

### Sample

The Avon Longitudinal Study of Parents and Children (ALSPAC) is a birth cohort that originally recruited pregnant women in what was previously the county of Avon, UK with expected dates of delivery 1st April 1991 to 31st December 1992. We included individuals from the initial enrolment sample: the initial number of pregnancies was 14 541. Of these, there were a total of 14 676 foetuses, resulting in 14 062 live births and 13 988 children who were alive at 1 year of age (Boyd et al., 2013; Fraser et al., 2012; Northstone et al., 2019). Twins are usually highly similar due to growing up in the same environment and therefore their data are not independent. Including twins in our study sample may therefore inflate any population-level association found. To mitigate this risk, we excluded the second-born of each twin pair. The study sample comprised of those who had data on any of the putative pubertal timing mediators (*N* = 6689): multiple imputation using chained equations was utilised to impute missing data on exposure, outcome and covariates. Data were collected via questionnaires and research clinics. The ALSPAC study website contains details of all the data that are available through a fully searchable data dictionary and variable search tool (http://www.bristol.ac.uk/alspac/researchers/our-data/).

Ethical approval for the study was obtained from the ALSPAC Ethics and Law Committee and the Local Research Ethics Committees.

### Measures

#### Childhood adversity

Nine domains of childhood adversity from birth to 9 years old were assessed using parent and child reports (Houtepen, Heron, Suderman, Tilling, & Howe, 2018; Russell et al., 2019). We assessed adversity up to age 9 to ensure that our exposure variable preceded onset of menarche (only 12 participants reported onset of menarche prior to age 9 in the study sample). These domains of adversity have been widely used in other studies of childhood adversity (Finkelhor, Shattuck, Turner, & Hamby, 2013; Hughes et al., 2017) and include physical, sexual or emotional abuse, parental mental health problems or suicide attempt, child being bullied, violence between parents, parental separation, parental criminal conviction and parental substance use (online Supplementary Table 1). Exposure to adversity was conceptualised as a continuous score of the number of different adversities experienced (range 0–9), as existing evidence shows a dose–response relationship between the number of types of adversity experienced and a range of negative health outcomes in later life (Anda et al., 2006; Hughes et al., 2017).

#### Self-harm

At age 16, young people provided self-reports of their lifetime history of self-harm in response to the question ‘Have you ever hurt yourself on purpose in any way (e.g. by taking an overdose of pills, or by cutting yourself)?’

#### Mediators: pubertal timing

We used age at peak height velocity (aPHV) and AAM as indicators of pubertal timing. aPHV is an objectively measured indicator of pubertal timing in both sexes that indicates the age at which the adolescent ‘growth spurt’ is at its most rapid (Demirjian, Buschang, Tanguay, & Patterson, 1985). The distribution of aPHV is normal in the general population and correlates well with other measures of pubertal timing (e.g. 0.79 with AAM in ALSPAC) (Demirjian et al., 1985; Marshall & Tanner, 1969, 1970). aPHV was calculated from multiple height measurements recorded at research clinics across childhood and adolescence using Superimposition by Translation and Rotation (SITAR), a mixed effects growth curve analysis described in detail elsewhere (Frysz, Howe, Tobias, & Paternoster, 2018).

AAM was assessed using data from nine postal questionnaires relating to pubertal development that were administered approximately annually from age 8 to 17. The questionnaires asked whether menstruation had started, and if so at what age (in years and months). The first-reported AAM was used to minimise recall error (Joinson, Heron, Araya, & Lewis, 2013).

#### Confounders and covariates

Participant sex, father absence during pregnancy, maternal education, housing tenure, material hardship and financial difficulties were treated as confounders of all paths in our models. We included body mass index (BMI) at age 9 as an intermediate confounder, because it could potentially be affected by childhood adversity and causally influence pubertal timing and self-harm.

#### Sensitivity analyses

We examined associations with four secondary dichotomous outcomes: lifetime history of self-harm with suicidal intent at age 16, multiple self-harm in past year (i.e. reported >1 episode) at age 16, self-harm at age 21 and lifetime history of self-harm with suicidal intent at age 21. We considered suicidal intent to be present if young people indicated that they ‘have ever seriously wanted to kill [themselves] on any occasion where [they] have hurt [themselves]’ or the ‘last time [they] hurt themselves it was because [they] wanted to die’. Self-harm and suicidal intent at age 21 were measured with the same questions asked at age 16. We conducted sensitivity analyses to explore whether effects were independent of psychopathology by excluding those with a psychiatric disorder assessed using the Development and Wellbeing Assessment (DAWBA) at age 15. DSM-IV diagnoses were generated using information from parents and young people via a computer algorithm (Goodman, Ford, Richards, Gatward, & Meltzer, 2000; Goodman, Heiervang, Collishaw, & Goodman, 2011). The primary results presented are from the imputed sample (*N* = 6698) with complete case results presented in the online Supplementary material.

### Analysis

We treated aPHV and AAM (in months) as continuous variables. We used multiple imputation by chained equations (separately for males and females; 50 imputations) in order to mitigate the potential for bias due to missing data (*N* = 6698), and compared this with results obtained from analyses in complete case data (*n* = 2373). Results were largely consistent across the imputed and complete case samples.

We conducted mediation analyses using structural equation modelling to implement Poisson regression (because self-harm was not a rare outcome) with robust standard errors, partitioning effects into direct and indirect effects using the products of coefficients approach (conducted using the *gsem* package in Stata v15). In complete case data, we used bootstrapping (*n* reps = 1000) to generate bias-corrected confidence intervals (CIs). In the aPHV model, there was no interaction between sex and aPHV (*p* > 0.1), therefore we included males and females in a single model, with sex as a covariate. We ran sensitivity analyses to explore whether findings were consistent when excluding those with psychiatric disorder at age 15, and for the secondary outcomes (self-harm with suicidal intent at age 16, multiple self-harm in past year at age 16, self-harm at age 21 and self-harm with suicidal intent at age 21).

## Results

### Description of sample

**Fig. 1.**
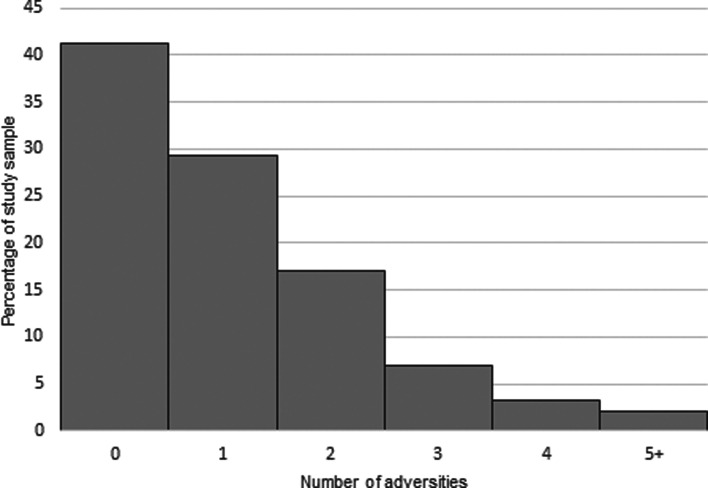
Number of ACEs per child (complete case data *N* = 2373).

Descriptive statistics for the imputed sample and complete cases are shown in Table 1. The study sample comprised of young people from more socioeconomically advantaged families compared with those not included in the study sample (online Supplementary Table 2). Of the nine adversities measured, parental mental health problems or suicide attempt were the most frequently experienced (39.3%), followed by parental separation and violence between parents (21.9% and 21.2% respectively; online Supplementary Tables 3 and 4). Figure 1 shows the number of adversities experienced across the sample, and online Supplementary Figs 1–4 show the distribution of pubertal timing measures by gender, as well as plots indicating the mean aPHV by number of ACEs and gender.

**Table 1.** Descriptive statistics: complete case and imputed sample

	Complete case (*N* = 2373)		Imputed sample (*N* = 6689, 50 imputed datasets)	
Continuous variables	Mean	s.d.	Mean	s.e.
Adversity score	1.08	1.23	1.38	0.0
AAM (months)	153.1	13.9	151.5	0.2
aPHV (males and females, months)	150.6	14.1	151.3	0.2
Age at PHV (females, months)	141.9	9.81	141.5	0.0
Age at PHV (males, months)	161.8	10.3	162.3	0.2
BMI age 9	17.5	2.7	17.8	0.2
Birthweight (g)	3369	655	3368	0.0
Maternal age at child birth (years)	29.93	4.26	29.0	8.0
Binary/categorical variables	*n*	%	%	s.e.
Psychiatric disorder at 15	87	4.1	6.59	0.4
Self-harm at 16	447	18.8	22.7	0.7
Self-harm at 21	372	21.8	23.7	0.7
Suicidal intent at 16	145	6.1	4.50	0.5
Suicidal intent at 21	202	8.5	6.53	0.6
Multiple self-harm at 16	242	10.5	14.6	0.6
Child sex (female)	1392	58.7	62.1	0.6
Housing tenure (not owned/mortgaged)	233	9.8	17.8	0.5
Maternal education				
Degree	590	24.9	16.5	0.5
A-level	731	30.8	26.7	0.5
GCSE	745	31.4	34.5	0.6
<GCSE	307	12.9	22.3	0.5
Material hardship	411	17.3	27.1	0.5
Equivalised household income (quintiles)				
Highest	724	30.5	22.7	0.5
	606	25.5	21.8	0.5
	482	20.3	20.5	0.5
	351	14.8	18.7	0.5
Lowest	210	8.85	16.3	0.6
Father absence during pregnancy	<5	<0.2	1.7	0.5
Maternal smoking during pregnancy (ever)	295	12.4	19.3	0.5
Parity				
0	1196	50.9	47.9	0.6
1	816	34.7	34.7	0.6
2	263	11.2	12.8	0.4
3+	75	3.2	4.6	0.3
White British ethnicity	2335	98.8	98.0	0.2

### Mediation results

There was robust evidence for direct effects of childhood adversity on risk of self-harm. For every additional type of adversity experienced; participants had on average a 12–14% increased risk of self-harm by age 16 (Table 2). The relative risk (RR) for the direct effect was 1.14 (95% CI 1.09–1.20) in the model with aPHV as the mediator, while in the model with AAM as the mediator the RR was 1.12 (95% CI 1.08–1.17). RRs were stronger for direct effects when outcomes were restricted to those who self-harmed with suicidal intent at 16 or 21 years. There was no evidence that pubertal timing (assessed using aPHV or AMM) mediated the association between childhood adversity and self-harm (indirect effect RR 1.00, 95% CI 1.00–1.00 for aPHV and RR 1.00, 95% CI 1.00–1.01 for AAM). On examining model results, we found evidence for associations between pubertal timing (aPHV and AAM) and self-harm, and between childhood adversity and self-harm. There was no evidence, however, of an association between childhood adversities and pubertal timing. RR estimates were marginally larger for complete case data, with wider CIs (online Supplementary Table 5).

**Table 2.** Results of mediation analyses assessing the relationship between number of adversities experienced by age 9, pubertal timing and later self-harm

Outcome	Mediator investigated	Direct effect			Indirect effect via mediator			Total effect		
		RR	95% CI	*p* value	RR	95% CI	*p* value	RR	95% CI	*p* value
Self-harm at 16	aPHV (both sexes, main analysis *N* = 6689)	1.14	1.09–1.20	<0.001	1.00	1.00–1.00	0.446	1.14	1.09–1.20	<0.001
Self-harm at 16	aPHV standardised by sex (both sexes, main analysis *N* = 6689)	1.14	1.09–1.19	<0.001	1.00	1.00–1.00	0.454	1.14	1.08–1.19	<0.001
Self-harm at 16	AAM (females *n* = 4040)	1.12	1.08–1.17	<0.001	1.00	1.00–1.01	0.224	1.12	1.08–1.17	<0.001
Self-harm at 16	aPHV (males only, *n* = 2533)	1.14	1.04–1.25	0.007	1.00	0.99–1.01	0.878	1.14	1.04–1.25	0.007
Self-harm at 16	aPHV (females only, *n* = 4156)	1.14	1.09–1.20	<0.001	1.00	0.99–1.00	0.273	1.14	1.09–1.20	<0.001

RR: relative risk; 95% CI: 95% confidence interval. RRs can be interpreted as the average percentage increase in risk (if >1) of the outcome for each additional type of adversity experienced prior to age 9 i.e. for row 1 direct effect, for every additional type of adversity experienced, participants were on average 14% more likely to report self-harm at age 16.

### Sensitivity analyses

In sensitivity analyses utilising outcomes of self-harm at age 21, self-harm with suicidal intent at ages 16 and 21, past-year multiple self-harm at age 16 and excluding those with psychiatric disorder at age 15, results for each mediator were consistent with the main findings (Tables 3 and 4). The direct effects were larger for self-harm with suicidal intent at 16 and 21 (aPHV model: RR at age 16 = 1.28, 95% CI 1.18–1.39; RR at age 21 = 1.20, 95% CI 1.12–1.28).

**Table 3.** Sensitivity analyses results of mediation analyses assessing the relationship between the number of adversities experienced by age 9, aPHV and later self-harm

Outcome	Mediator	Direct effect			Indirect effect via mediator			Total effect		
		RR	95% CI	*p* value	RR	95% CI	*p* value	RR	95% CI	*p* value
Self-harm with suicidal intent at 16	aPHV (both sexes *N* = 6689)	1.28	1.18–1.39	<0.001	1.00	1.00–1.00	0.496	1.28	1.18–1.39	<0.001
Multiple self-harm in past year at 16	aPHV (both sexes *N* = 6689)	1.12	1.05–1.20	0.001	1.00	1.00–1.00	0.504	1.12	1.04–1.19	0.001
Self-harm at 16; no psychiatric disorder at 15	aPHV (both sexes *n* = 6535)	1.13	1.08–1.19	<0.001	1.00	1.00–1.00	0.442	1.13	1.08–1.19	<0.001
Self-harm at 21	aPHV (both sexes *N* = 6689)	1.13	1.08–1.18	<0.001	1.00	1.00–1.00	0.572	1.13	1.08–1.18	<0.001
Self-harm with suicidal intent at 21	aPHV (both sexes *N* = 6689)	1.20	1.12–1.28	<0.001	1.00	1.00–1.00	0.5	1.20	1.12– 1.28	<0.001

RR: relative risk; 95% CI: 95% confidence interval. RRs can be interpreted as the average percentage increase in risk (if >1) of the outcome for each additional type of adversity experienced prior to age 9 i.e. for row 1 direct effect, for every additional type of adversity experienced, participants were on average 28% more likely to report self-harm with suicidal intent at age 16.

**Table 4.** Sensitivity analyses results of mediation analyses assessing the relationship between the number of adversities experienced by age 9, AAM and later self-harm

Outcome	Mediator	Direct effect			Indirect effect via mediator			Total effect		
		RR	95% CI	*p* value	RR	95% CI	*p* value	RR	95% CI	*p* value
Self-harm with suicidal intent at 16	AAM (females *n* = 4040)	1.21	1.12–1.29	<0.001	1.00	1.00–1.01	0.229	1.21	1.13–1.30	<0.001
Multiple self-harm in past year at 16	AAM (females *n* = 4040)	1.13	1.06–1.20	<0.001	1.00	1.00–1.01	0.221	1.13	1.07–1.21	<0.001
Self-harm at 16; no psychiatric disorder at 15	AAM (females *n* = 3844)	1.12	1.07–1.17	<0.001	1.00	1.00–1.01	0.169	1.12	1.07–1.17	<0.001
Self-harm at 21	AAM (females *n* = 4040)	1.09	1.05–1.14	<0.001	1.00	1.00–1.00	0.312	1.10	1.05–1.14	<0.001
Self-harm with suicidal intent at 21	AAM (females *n* = 4040)	1.17	1.09–1.24	<0.001	1.00	1.00–1.01	0.225	1.17	1.10–1.25	<0.001

RR: relative risk; 95% CI: 95% confidence interval. RRs can be interpreted as the average percentage increase in risk (if >1) of the outcome for each additional type of adversity experienced prior to age 9 i.e. for row 1 direct effect, for every additional type of adversity experienced, participants were on average 21% more likely to report self-harm with suicidal intent at age 16.

## Discussion

We found that childhood adversity and early timing of puberty are independent risk factors for later self-harm in both males and females. There was no evidence in this study for an association between total childhood adversities and pubertal timing, and no mediating effect of early pubertal timing on the relationship between childhood adversity and self-harm. Our findings are consistent with a large study (*n* = 14 000 adolescents) that found no evidence for a mediating effect of pubertal timing on the relationship between childhood adversity and depressive symptoms in adolescence (Strong, Tsai, Lin, & Cheng, 2016). Just over one-in-five young people in our sample reported self-harm at age 16 (18.8% in complete case data and 22.7% in the imputed data). This is higher than the prevalence of self-harm in adolescents aged 13–18 in the community reported in a recent meta-analysis (16.9%, 95% CI 15.1–18.9), although estimates vary across samples due to differences in population and methodology (Gillies et al., 2018). The prevalence of ACEs in our sample (58.7% reported at least one ACE) was consistent with estimates from a recent meta-analysis of over 250 000 participants and in a similar UK-based cohort assessing ACEs from age 0 to 5 years (57% and 50% respectively reported at least once ACE), while 5.0% in our sample reported four or more ACEs, compared with 13% with at least four ACEs in the meta-analysis and 1.4% in the Millennium Cohort (Hughes et al., 2017; Straatmann et al., 2020). We measured ACEs between the ages of 0 and 9 years whereas the meta-analysis largely includes data from adult samples. Differences in socioeconomic disadvantage across populations might also explain the difference in those accumulating more than four ACEs (Straatmann et al., 2020). Our estimates of the distribution of AAM and peak height velocity are also in line with other studies (e.g. Granados, Gebremariam, & Lee, 2015; Parent et al., 2003).

There are currently fewer studies of adversity and pubertal timing in boys than girls, although research in this area is increasing (Hawton et al., 2003; Lei et al., 2018; Mendle, 2014; Zhang et al., 2019). Existing studies reporting null findings may also be subject to publication bias. Regarding females, our study found no association between cumulative adversity and AAM. A study of the ALSPAC mothers' cohort found that only sexual abuse was associated with earlier menarche (Magnus et al., 2018). One study found evidence for differing dose–response relationships between several forms of abuse with AAM, with sexual and physical abuse predicting earlier AAM, and physical abuse also being associated with later AAM (Boynton-Jarrett et al., 2013). Exposure to specific types of adversity may also impact differentially on physiological development during puberty, with findings for sexual abuse being particularly strong (Zabin, Emerson, & Rowland, 2005). Puberty and developmental maturational processes are largely controlled by biological systems, however there is evidence that environmental factors such as diet, nutrition and the presence of a step-father in the household (Zabin et al., 2005) contribute to pubertal timing. We controlled for BMI and father absence during pregnancy, however there may be other factors that were not accounted for in our models.

### Strengths and limitations

We utilised data from a large sample of young people, who are broadly representative of the UK population although slightly more socioeconomically advantaged and less ethnically diverse. The most disadvantaged participants were more likely to drop-out (Wolke et al., 2009), but our findings were similar across imputed and complete case analyses. The longitudinal nature of the mediation analysis is a key strength of the study design, allowing us to draw inferences about the direction of effects with measures of adversity occurring before puberty, and pubertal timing prior to measures of self-harm. We used an objective measure of pubertal timing, aPHV, as our primary mediator. This was based on repeated measures of height taken during research clinics, rather than self-reported height, and allowed us to investigate associations for males as well as females. Males are underrepresented in research on puberty, and much of the existing studies that link adversity and puberty, and puberty and self-harm, are based on females. Our estimates of prevalence of ACEs and self-harm were in line with other studies, which gives confidence in the potential generalisability of our findings.

There are limitations to our study; there may be bias in reporting of adversity, as much of the information was collected from questionnaires to parents. The outcome data are now 10 years old, and rates of self-harm are now higher than 10 years ago (McManus et al., 2019; Sadler et al., 2020). Parents may under-report adversities due to perceived stigma or social desirability. However, estimates of adversities in ALSPAC are in line with similar studies. Similarly, self-harm was self-reported by individuals, and the prevalence is similar to other non-clinical samples. We did not account for the severity of adversities in our model, beyond including multiple forms of adversity. It is likely that exposure to severe and repeated adversity or multiple adversities will have differential impacts on pubertal timing, compared with brief exposure to one adversity. This was not adequately captured by our measures and future work could explore this further. We utilised a cumulative index of number of adversities experienced, which is common in the scientific literature as exposure to a range of ACEs has been shown to have associations with a range of health outcomes across the lifecourse (Hughes et al., 2017) and because ACEs do not often occur in isolation it is difficult to disentangle the impact of a single ACE on a single outcome.

Our sample was more socioeconomically advantaged and less ethnically diverse than the UK population. Socioeconomic disadvantage is associated with a higher burden of ACEs in studies from a range of countries (Straatmann et al., 2020; Walsh, McCartney, Smith, & Armour, 2019). In our sample this may mean that exposure to ACEs was underestimated and may explain why we have a smaller proportion of participants with four or more ACEs compared with other studies (Hughes et al., 2017) and therefore associations between adversities and self-harm would be attenuated towards the null. To some extent, this should be accounted for by our including several indicators of socioeconomic disadvantage as covariates in our models. Interestingly, studies have found that adversity confers additional risk of negative mental health outcomes after accounting for socioeconomic disadvantage and ethnicity of participants (Nurius, Logan-Greene, & Green, 2012), as seen in our findings also. There is also evidence that ethnic background or race may be differentially associated with likelihood of experiencing adversity. Most data on this come from the USA (e.g. Putnam-Hornstein, Needell, King, & Johnson-Motoyama, 2013) however studies based in UK populations have shown that ACEs were lower in those of Asian ethnicity, while those who were non-white and non-Asian had higher levels of adversity (Bellis, Hughes, Leckenby, Perkins, & Lowey, 2014). Our analysis may therefore either under or over-estimate associations between ACEs and self-harm in those with ethnic backgrounds that are not well represented in ALSPAC.

### Implications

Further research should also explore the relationship between the severity and timing of exposure to adversity, and the role of specific types of adversity, on pubertal timing in both males and females in order to extend current understanding of how this relationship may impact on future health and wellbeing. Given the recent evidence for an increasing rate of self-harm in adolescence (Sadler et al., 2020), and the relationship with childhood adversity, research is needed to understand mechanisms that explain why adverse exposures in childhood increase the risk of self-harm. Identifying modifiable mediators could inform the development of prevention and intervention efforts. Adversity is known to have multiple impacts on development, and mediating mechanisms could include cognitive, behavioural, emotional, social and biological factors (Dube et al., 2003). Potential mediators that could be studied further include mental health problems (Enns et al., 2006) adolescent drinking and drug use (Dube et al., 2001; Turecki, Ernst, Jollant, Labonté, & Mechawar, 2012), family support (Cassels et al., 2018), immune functioning and stress reactivity (Berens, Jensen, & Nelson, 2017). Biological mechanisms may also include dysregulated stress-response systems, or cognitive deficits that impact ability to regulate behaviour and emotions (Turecki et al., 2012). As with many complex traits, it is likely that there are multiple interacting causal pathways from adversity to self-harm.

## Conclusions

Our findings suggest that both childhood adversity and early pubertal timing are risk factors for later self-harm in both males and females. However, we found no evidence that the effect of adversity on self-harm is mediated through earlier pubertal timing. There is a need to identify mechanisms that explain why childhood adversity is associated with an increased risk of self-harm in order to inform the development of prevention and intervention efforts. School-based and psychosocial interventions targeting adolescent risky behaviours and social adjustment, as well as improved quality of puberty education in schools, are needed. Interventions should be targeted prior to and during pubertal onset, given that those who enter puberty earliest are at highest risk of later self-harm.

## Supplementary material

For supplementary material accompanying this paper visit https://doi.org/10.1017/S0033291721000611. click here to view supplementary material
