# Supplementary material for: Childhood adversity, pubertal timing and self-harm: a longitudinal cohort study
Source: Psychol Med. 2021 Mar 8;52(16):3807–15. doi: 10.1017/S0033291721000611 (PMC9811347; doi:10.1017/S0033291721000611)
Supplement: Supplementary file 1 [file S0033291721000611sup001.docx]

# Childhood adversity, pubertal timing and self-harm: a longitudinal cohort study: supplementary material

Abigail Russell et al. Psychological Medicine 2021

## Supplementary Table 1. Adverse Childhood Experiences (ACEs) definitions

| **ACE** | **Definition** | **Number of questions** | **Age range of questions asked** | **Respondents** |
| --- | --- | --- | --- | --- |
| Sexual abuse | Was the child sexually abused | 7 | 18 months to age 9 | Mother |
| Physical abuse | Whether physically cruel to child | 31 | 8 weeks to 9 years | Mother and Partner |
| Emotional abuse | Whether or not mum/partner had been emotionally cruel to the child | 32 | 8 months to 9 years | Mother and Partner |
| Parent substance use | Daily use of cannabis or any use of other drugs. Or, alcohol problem by self-reported problematic use, and saw a doctor because of it | 62 | 8 weeks to 9 years | Mother and Partner |
| Parent mental health problems or suicide attempt | Depression scores (EPDS>12) and medication, presence of schizophrenia, bulimia, anorexia or attempted suicide. | 57 | 8 weeks to 9 years | Mother and Partner |
| Violence between parents | Parent experienced physical cruelty from partner, or displayed (specific types) of violence towards partner | 43 | 8 weeks to 9 years | Mother and Partner |
| Parental separation | Parents divorced or separated. Degree to which this impacted on the child. | 32 | 8 weeks to 9 years | Mother and Partner |
| Bullying | Child bullied | 6 | 8 years-8.5 years | Child |
| Parent convicted | Parent convicted off offence | 18 | 8 weeks to 9 years | Mother and Partner |

Notes: ACE adverse childhood experience. EPDS Edinburgh Postnatal Depression Scale. Several of the ACEs included questions about how much the ACE had impacted on the parent or child.

## Supplementary Table 2. Descriptive statistics: sample for imputation and ALSPAC

|  | **Sample for imputation (n=6689)** | | **Rest of ALSPAC (n=7099)** | | |
| --- | --- | --- | --- | --- | --- |
| **Description** | **mean** | **SD** | **mean** | **SD** | **P** |
| Adversity score | 1.15 | 1.28 | 1.00 | 1.32 | 0.036 |
| Age at menarche (months) | 151.5 | 14.10 |  |  |  |
| Age at peak height velocity (months) | 151.3 | 14.43 |  |  |  |
| Age at PHV (females, months) | 141.5 | 9.82 |  |  |  |
| Age at PHV (males, months) | 162.3 | 10.26 |  |  |  |
| BMI age 9 | 17.7 | 2.87 | 17.8 | 3.05 | 0.2312 |
| Birthweight (g) | 3369 | 657.6 | 3351 | 673.38 | 0.1237 |
| Maternal age at child birth | 29.0 | 4.62 | 27.0 | 5.10 | <0.001 |
|  | **n** | **%** | **n** | **%** |  |
| Psychiatric disorder at 15 | 324 | 6.59 | 13.0 | 8.72 | 0.302 |
| Self-harm at 16 | 837 | 19.7 | 68 | 12.19 | <0.001 |
| Self-harm at 21 | 766 | 21.9 | 77 | 14.10 | <0.001 |
| Suicidal intent at 16 | 301 | 7.09 | 25 | 4.48 | 0.021 |
| Suicidal intent at 21 | 437 | 8.95 | 49 | 5.63 | 0.001 |
| Multiple self-harm at 16 | 448 | 10.8 | 39 | 7.04 | 0.006 |
| Child sex (female) | 4156 | 62.1 | 2517 | 35.5 | <0.001 |
| Housing tenure (not owned/mortgaged) | 1119 | 17.3 | 2312 | 36.2 | <0.001 |
| Maternal education |  |  |  |  |  |
| *Degree* | 1083 | 16.8 | 493 | 8.49 | <0.001 |
| *A-level* | 1735 | 26.9 | 1022 | 17.6 |  |
| *GCSE* | 2231 | 34.6 | 2007 | 34.54 |  |
| *<GCSE* | 1393 | 21.6 | 2288 | 39.38 |  |
| Material hardship | 1812 | 27.1 | 3149 | 44.36 | <0.001 |
| Equivalised household income (quintiles) |  |  |  |  |  |
| *Highest* | 1380 | 23.4 | 611 | 15.59 | <0.001 |
|  | 1302 | 22.1 | 658 | 16.79 |  |
|  | 1216 | 20.7 | 734 | 18.73 |  |
|  | 1087 | 18.5 | 857 | 21.87 |  |
| *Lowest* | 905 | 15.4 | 1058 | 27.00 |  |
| Father absence during pregnancy | 102 | 1.6 | 245 | 3.81 | <0.001 |
| Maternal smoking during pregnancy (ever) | 1268 | 19.1 | 2274 | 33.87 | <0.001 |
| Parity |  |  |  |  |  |
| *0* | 3087 | 47.8 | 2625 | 41.68 | <0.001 |
| *1* | 2249 | 34.8 | 2217 | 35.20 |  |
| *2* | 830 | 12.9 | 992 | 15.75 |  |
| *3+* | 293 | 4.5 | 464 | 7.37 |  |
| White British ethnicity | 6303 | 98.1 | 5543 | 96.55 | <0.001 |

## Supplementary Table 3. Adversities experienced by sample (sample for imputation and rest of ALSPAC)

|  | **Sample for imputation (n=6689)** | | **Rest of ALSPAC (n=7099)** | | **p** |
| --- | --- | --- | --- | --- | --- |
|  | **n** | **%** | **n** | **%** |  |
| Sexual abuse | 35 | 0.58 | 16 | 0.46 | 0.453 |
| Physical abuse | 347 | 6.33 | 168 | 6.53 | 0.73 |
| Emotional abuse | 795 | 16.2 | 405 | 18.1 | 0.048 |
| Child experiences bullying | 592 | 11.3 | 194 | 13.9 | 0.006 |
| Violence between parents | 848 | 17.7 | 322 | 20.4 | 0.019 |
| Parent substance use | 459 | 8.65 | 237 | 10.3 | 0.021 |
| Parent mental health problems or suicide | 1967 | 36.5 | 965 | 40.9 | <0.001 |
| Parent criminal conviction | 343 | 6.17 | 183 | 6.67 | 0.376 |
| Parental separation | 939 | 17.9 | 542 | 24.2 | <0.001 |

## Supplementary Table 4. Adversities experienced by sample (complete case and imputed data)

|  | **COMPLETE CASE (n=2373)** | | **Imputed sample (N=6689, 50 iterations)** | |
| --- | --- | --- | --- | --- |
|  | **n** | **%** | **Percentage** | **95% CI** |
| Sexual abuse | 9 | 0.38 | 0.82 | 0.55, 1.08 |
| Physical abuse | 153 | 6.45 | 6.98 | 6.23, 7.72 |
| Emotional abuse | 350 | 14.75 | 17.73 | 16.4, 19.0 |
| Child experiences bullying | 246 | 10.37 | 11.81 | 11.0, 12.7 |
| Violence between parents | 392 | 16.52 | 21.18 | 20.0, 22.4 |
| Parent substance use | 178 | 7.5 | 10.92 | 9.96, 11.9 |
| Parent mental health problems or suicide | 797 | 33.59 | 39.29 | 37.9, 40.7 |
| Parent criminal conviction | 137 | 5.77 | 6.89 | 6.19, 7.59 |
| Parental separation | 298 | 12.56 | 21.90 | 20.7, 23.1 |

## Supplementary Figure 1. Age at peak height velocity in males in study sample

**

## Supplementary Figure 2. Age at peak height velocity in females in study sample

**

## Supplementary Figure 3. Age at menarche in females in study sample

**

## Supplementary Figure 4. Descriptive chart showing mean age at peak height velocity for males and females by number of adversities experienced

## Supplementary Table 5 Complete case mediation results

| Outcome | Model | Direct effect | | | Indirect effect via mediator | | | Total effect | | |
| --- | --- | --- | --- | --- | --- | --- | --- | --- | --- | --- |
|  |  | RR | 95% CI | p value | RR | 95% CI | p value | RR | 95% CI | p value |
| Self-harm at 16 | Age at peak height velocity (both sexes, main analysis N=2251) | 1.16 | 1.09, 1.24 | <0.001 | 1.00 | 0.99, 1.00 | 0.508 | 1.15 | 1.09, 1.24 | <0.001 |
| Self-harm at 16 | Age at menarche (females n=1380) | 1.16 | 1.09, 1.23 | <0.001 | 1.00 | 0.99, 1.01 | 0.767 | 1.16 | 1.09, 1.23 | <0.001 |
|  |  |  |  |  |  |  |  |  |  |  |
| Self-harm at 16 | Age at peak height velocity (males only, n=981) | 1.18 | 1.04, 1.38 | 0.015 | 1.00 | 0.97, 1.01 | 0.765 | 1.18 | 1.04, 1.38 | 0.018 |
| Self-harm at 16 | Age at peak height velocity (females only, n=1270) | 1.15 | 1.08, 1.23 | <0.001 | 1.00 | 0.99, 1.00 | 0.611 | 1.15 | 1.08, 1.23 | <0.001 |
|  |  |  |  |  |  |  |  |  |  |  |
| **Sensitivity analyses- age at peak height velocity** | |  |  |  |  |  |  |  |  |  |
| Self-harm with suicidal intent at 16 | Age at peak height velocity (both sexes N=3006) | 1.28 | 1.13, 1.41 | <0.001 | 1.00 | 0.99, 1.00 | 0.942 | 1.28 | 1.13, 1.41 | <0.001 |
| Multiple self-harm in past year at 16 | Age at peak height velocity (both sexes N=2251) | 1.10 | 1.01, 1.20 | 0.025 | 1.00 | 0.99, 1.00 | 0.662 | 1.10 | 1.01, 1.20 | 0.026 |
| Self-harm at 16; no psychiatric disorder at 15 | Age at peak height velocity (both sexes n=2873) | 1.17 | 1.09, 1.23 | <0.001 | 1.00 | 0.99, 1.00 | 0.661 | 1.17 | 1.09, 1.23 | <0.001 |
| Self-harm at 21 | Age at peak height velocity (both sexes N=3006) | 1.12 | 1.06, 1.20 | <0.001 | 1.00 | 1.00, 1.00 | 0.957 | 1.12 | 1.06, 1.20 | <0.001 |
| Self-harm with suicidal intent at 21 | Age at peak height velocity (both sexes, main analysis N=3705) | 1.25 | 1.13, 1.35 | <0.001 | 1.00 | 1.00, 1.00 | 0.931 | 1.25 | 1.14, 1.35 | <0.001 |
|  |  |  |  |  |  |  |  |  |  |  |
| **Sensitivity analyses- age at menarche** | |  |  |  |  |  |  |  |  |  |
| Self-harm with suicidal intent at 16 | Age at menarche (females n=1762) | 1.28 | 1.14, 1.44 | <0.001 | 1.00 | 0.99, 1.02 | 0.629 | 1.28 | 1.14, 1.44 | <0.001 |
| Multiple self-harm in past year at 16 | Age at menarche (females n=1380) | 1.15 | 1.05, 1.27 | 0.002 | 1.00 | 0.99, 1.01 | 0.779 | 1.15 | 1.05, 1.27 | 0.002 |
| Self-harm at 16; no psychiatric disorder at 15 | Age at menarche (females n=1681) | 1.17 | 1.10, 1.25 | <0.001 | 1.00 | 1.00, 1.00 | 0.583 | 1.18 | 1.10, 1.26 | <0.001 |
| Self-harm at 21 | Age at menarche (females n=1762) | 1.14 | 1.07, 1.22 | <0.001 | 1.00 | 1.00, 1.01 | 0.654 | 1.14 | 1.07, 1.22 | <0.001 |
| Self-harm with suicidal intent at 21 | Age at menarche (females n=1762) | 1.24 | 1.12, 1.36 | <0.001 | 1.00 | 0.99, 1.01 | 0.617 | 1.24 | 1.12, 1.37 | <0.001 |
